# Supplementary material for: The MAGMA pipeline for comprehensive genomic analyses of clinical Mycobacterium tuberculosis samples
Source: PLoS Comput Biol. 2023 Nov 29;19(11):e1011648. doi: 10.1371/journal.pcbi.1011648 (PMC10686480; doi:10.1371/journal.pcbi.1011648)

**Figure A:** Phylogenetic tree created by (a) MAGMA including complex regions (IncComplex)


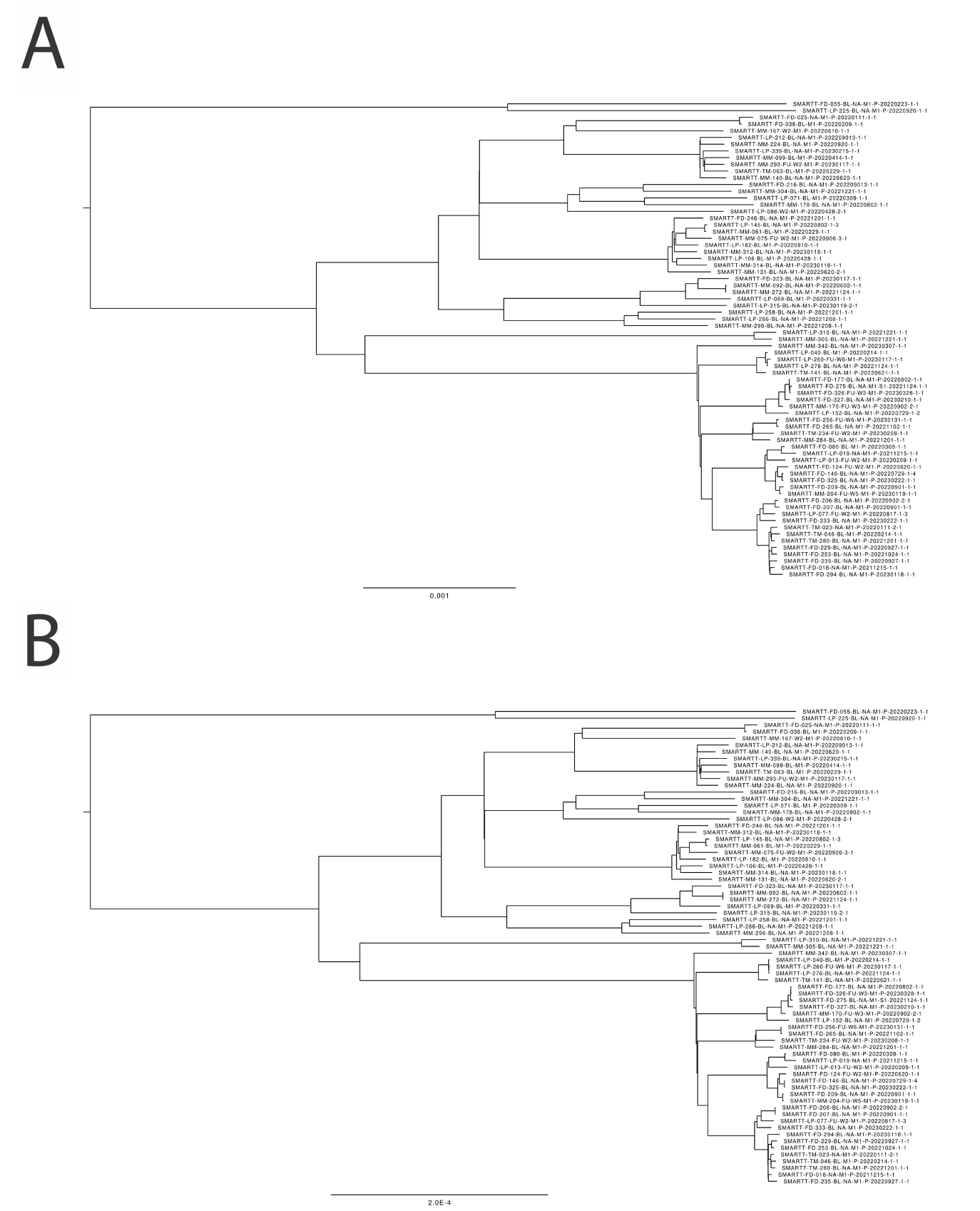


**Figure B:** Phylogenetic tree created by MAGMA excluding complex regions (ExComplex)


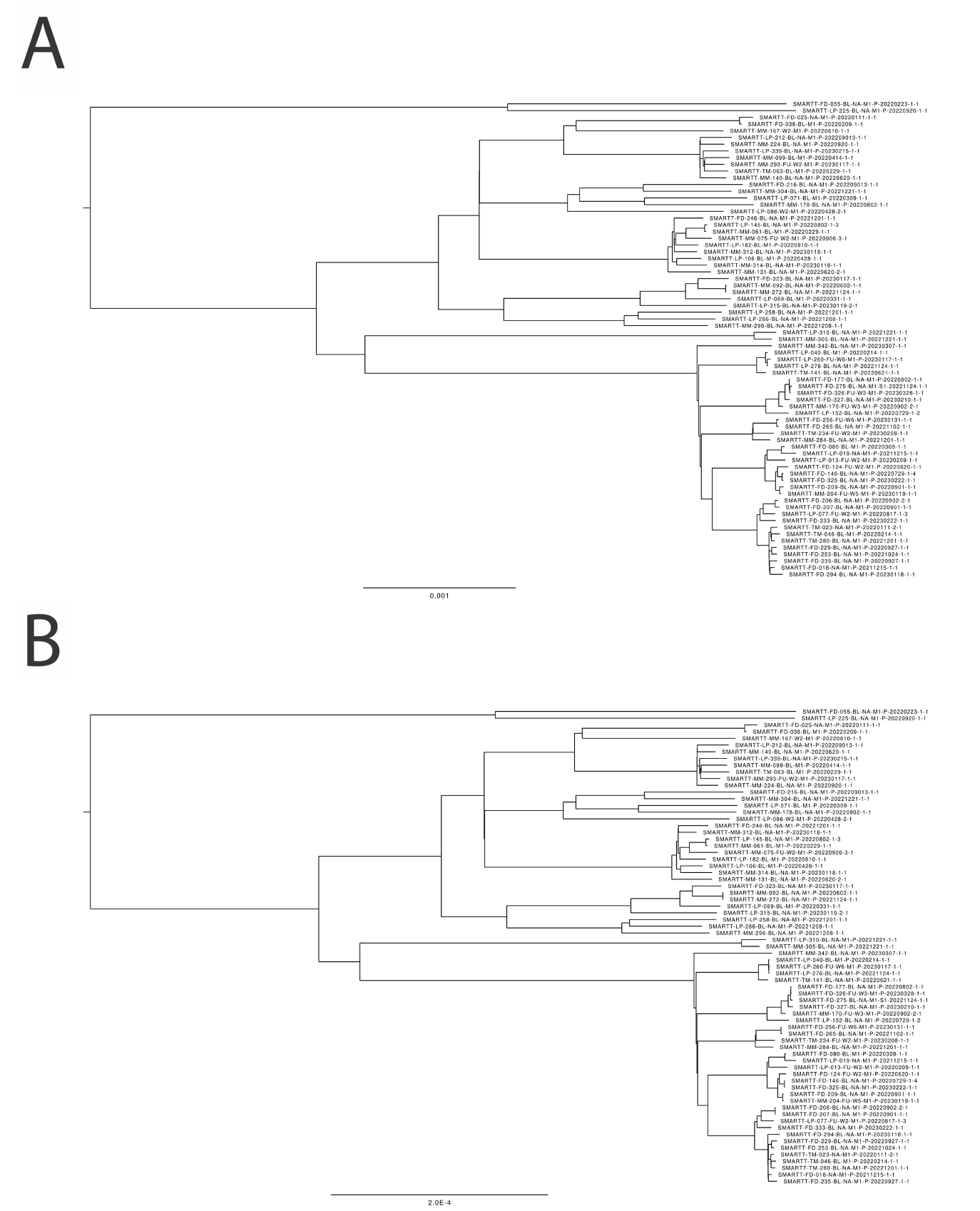


**Figure C:** Phylogenetic tree created by MAGMA excluding complex regions


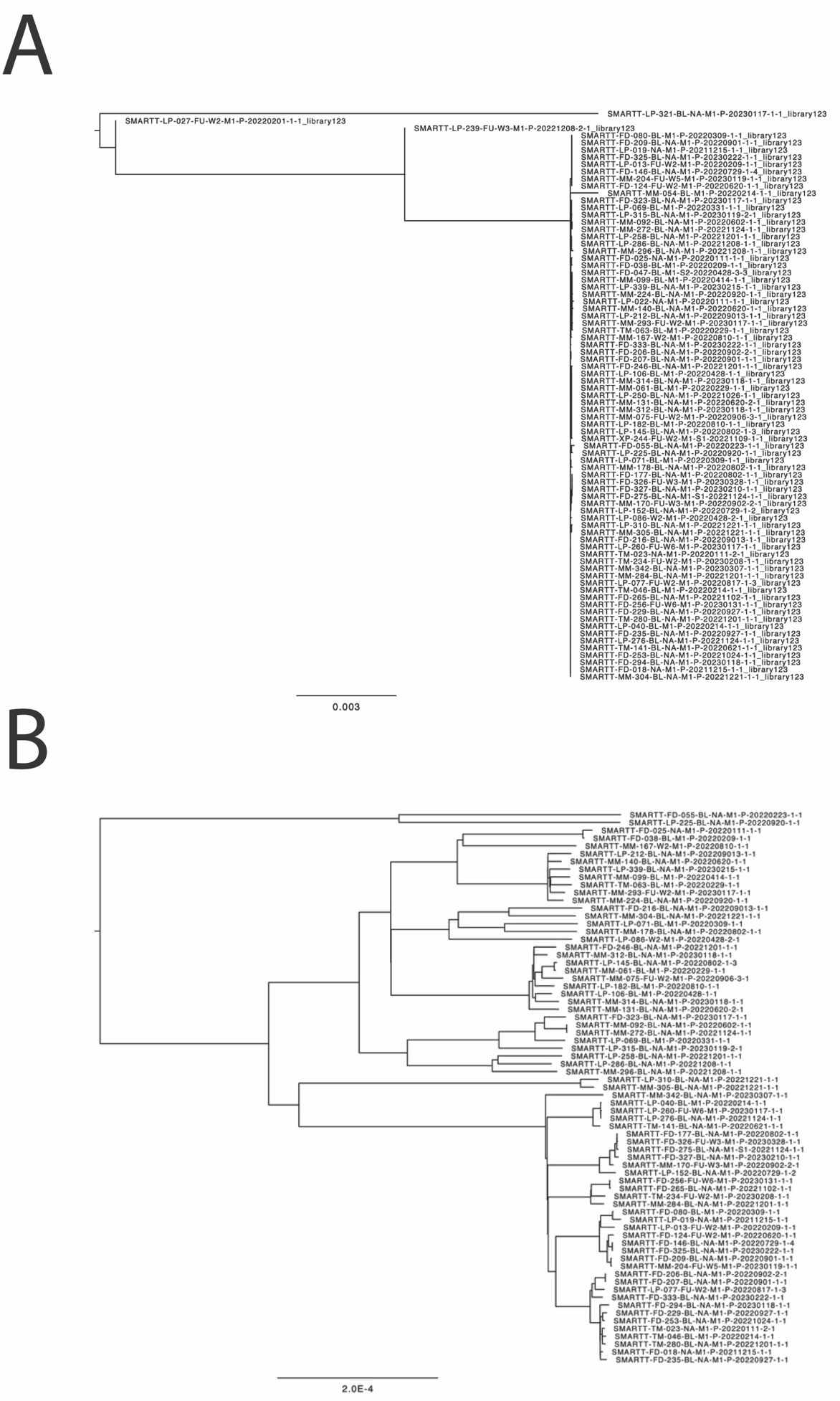


**Figure D:** Phylogenetic tree created by unfiltered MTBseq


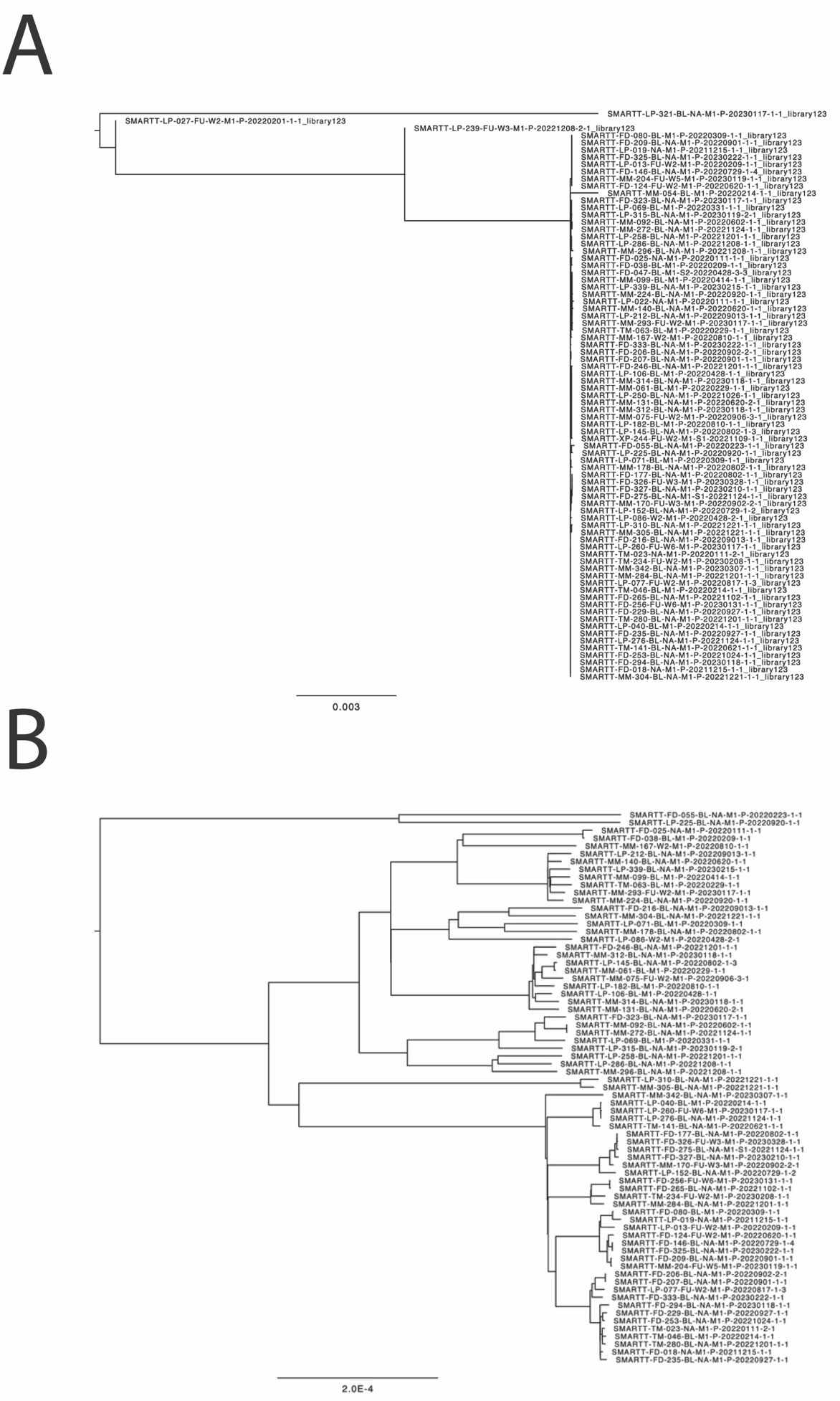


**Figure E:** Face to face comparison of the ExComplex MAGMA (right) and filtered MTBseq tree (left)


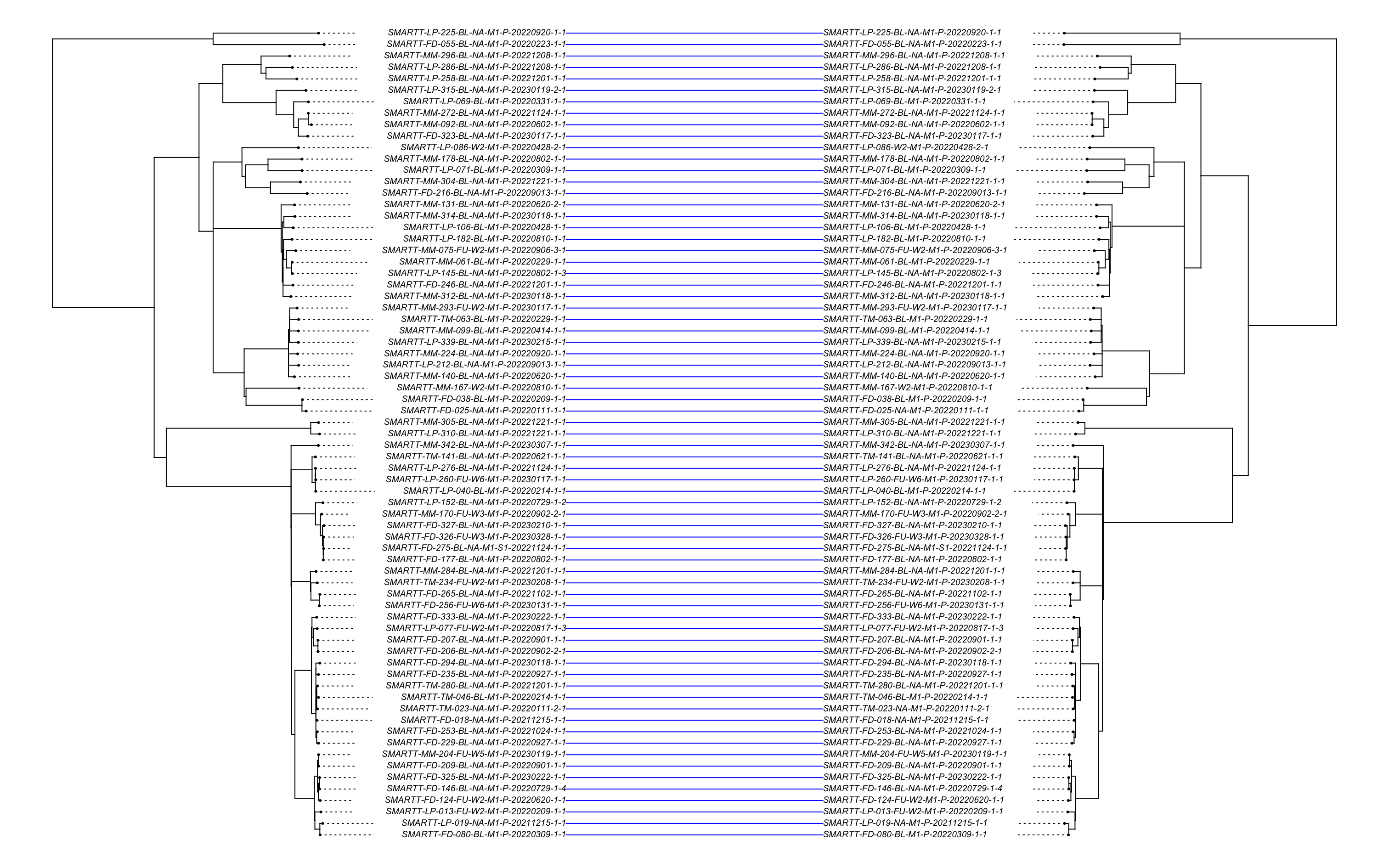


**Figure F:** Heatmap of the MAGMA phylogenetic tree excluding complex region


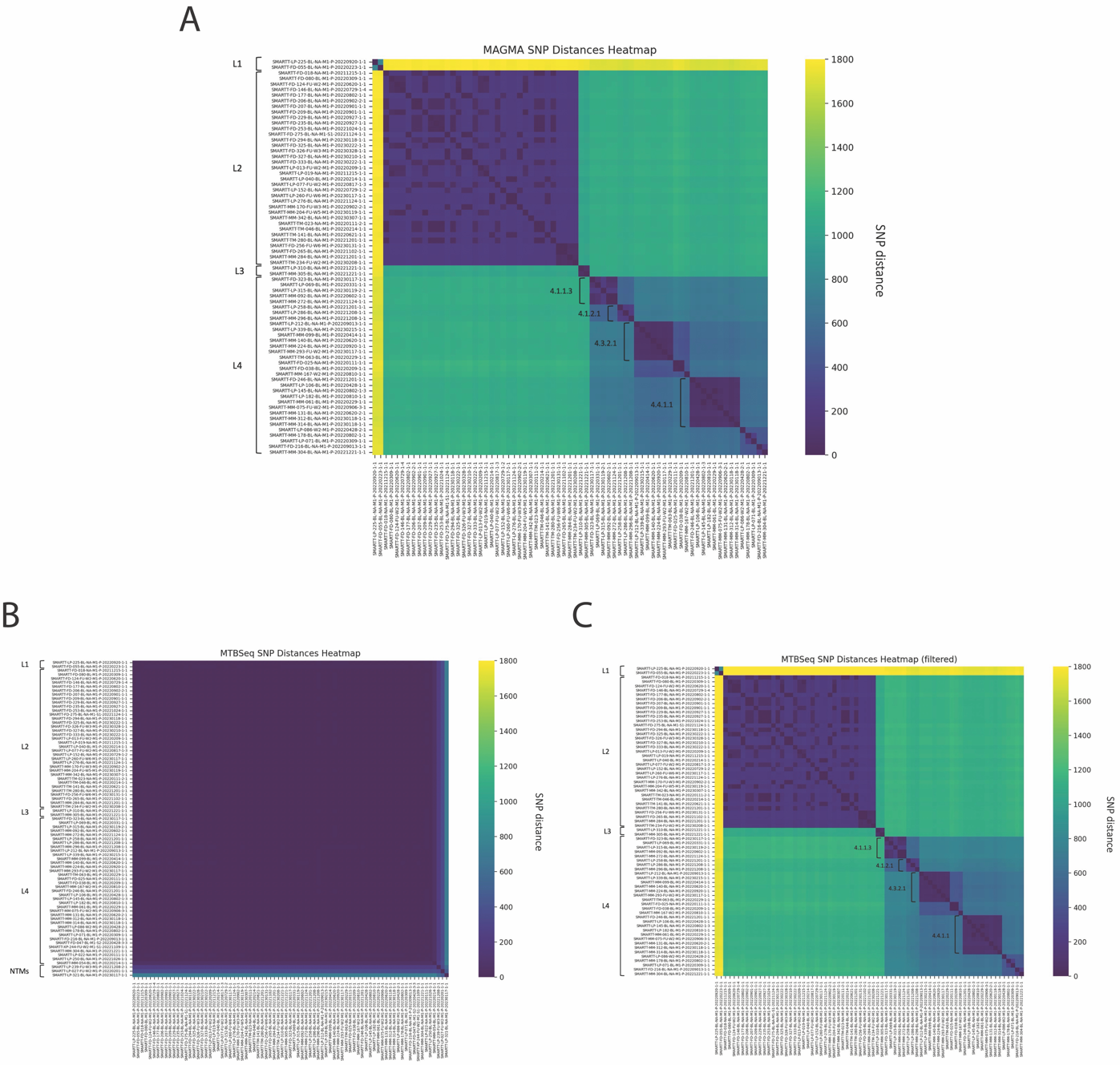


**Figure G:** Heatmap of default MTBseq tree


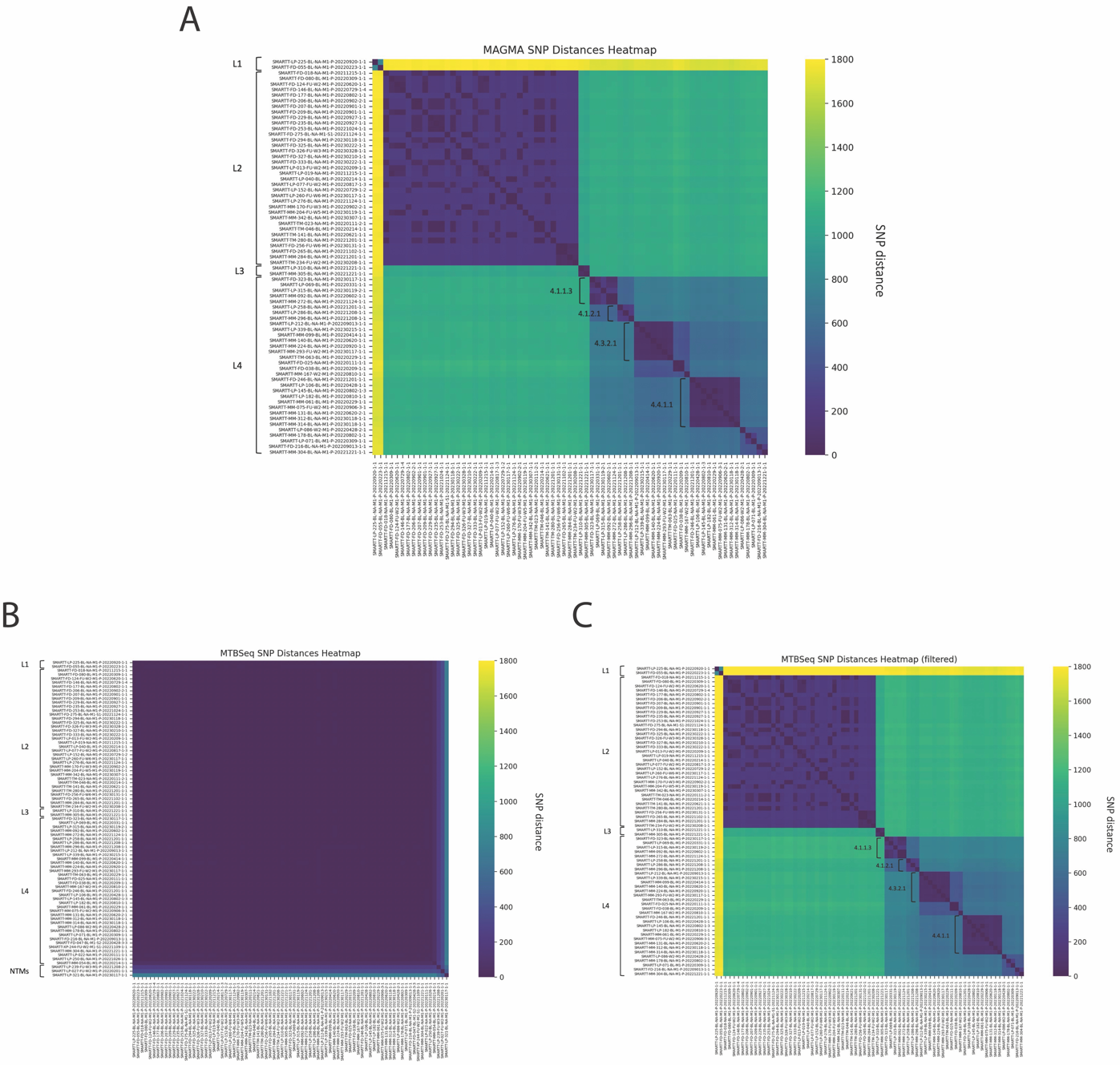


**Figure H:** Heatmap of the filtered MTBseq tree


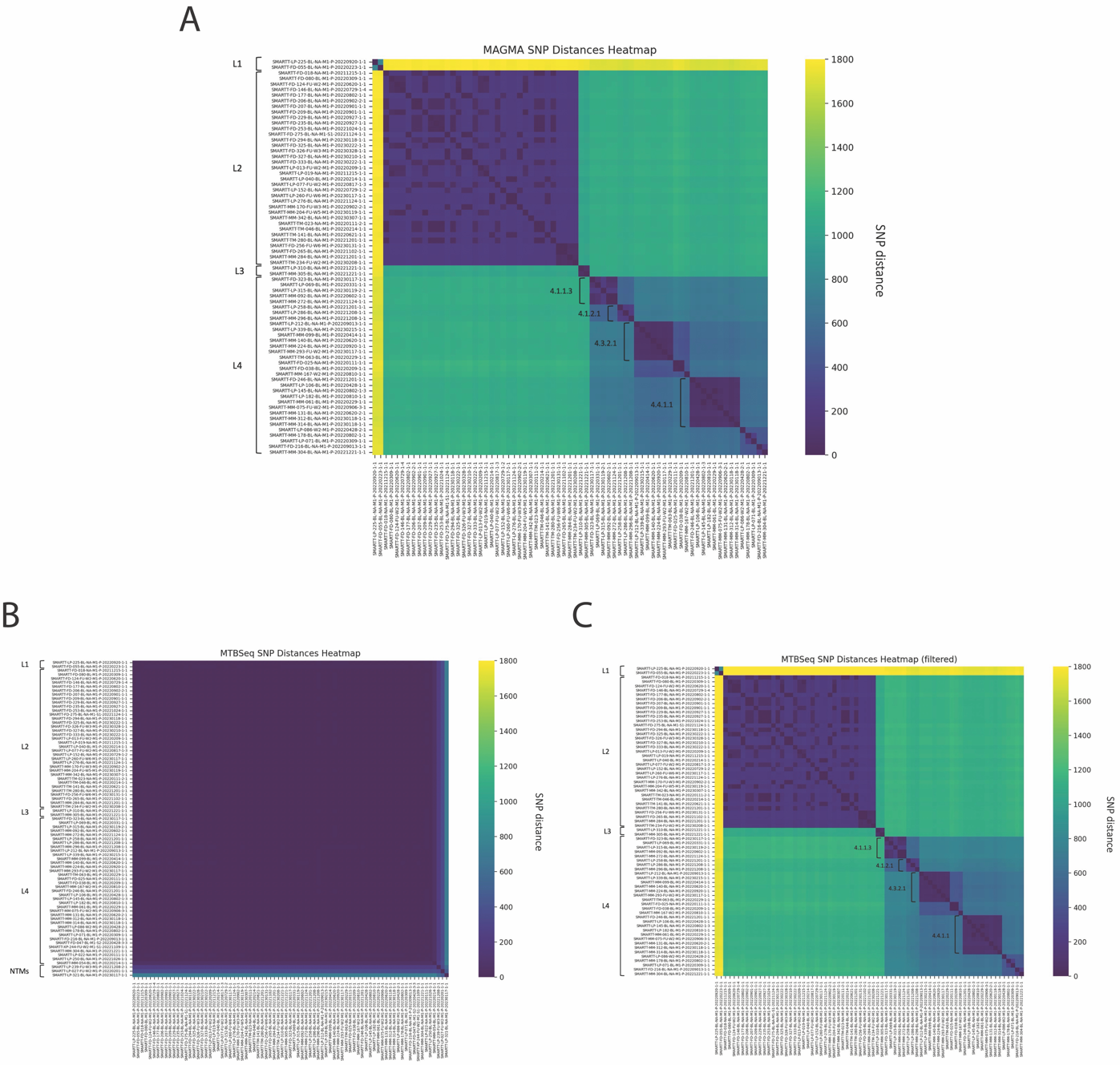


**Figure I:** Quality Control and Mapping Workflow


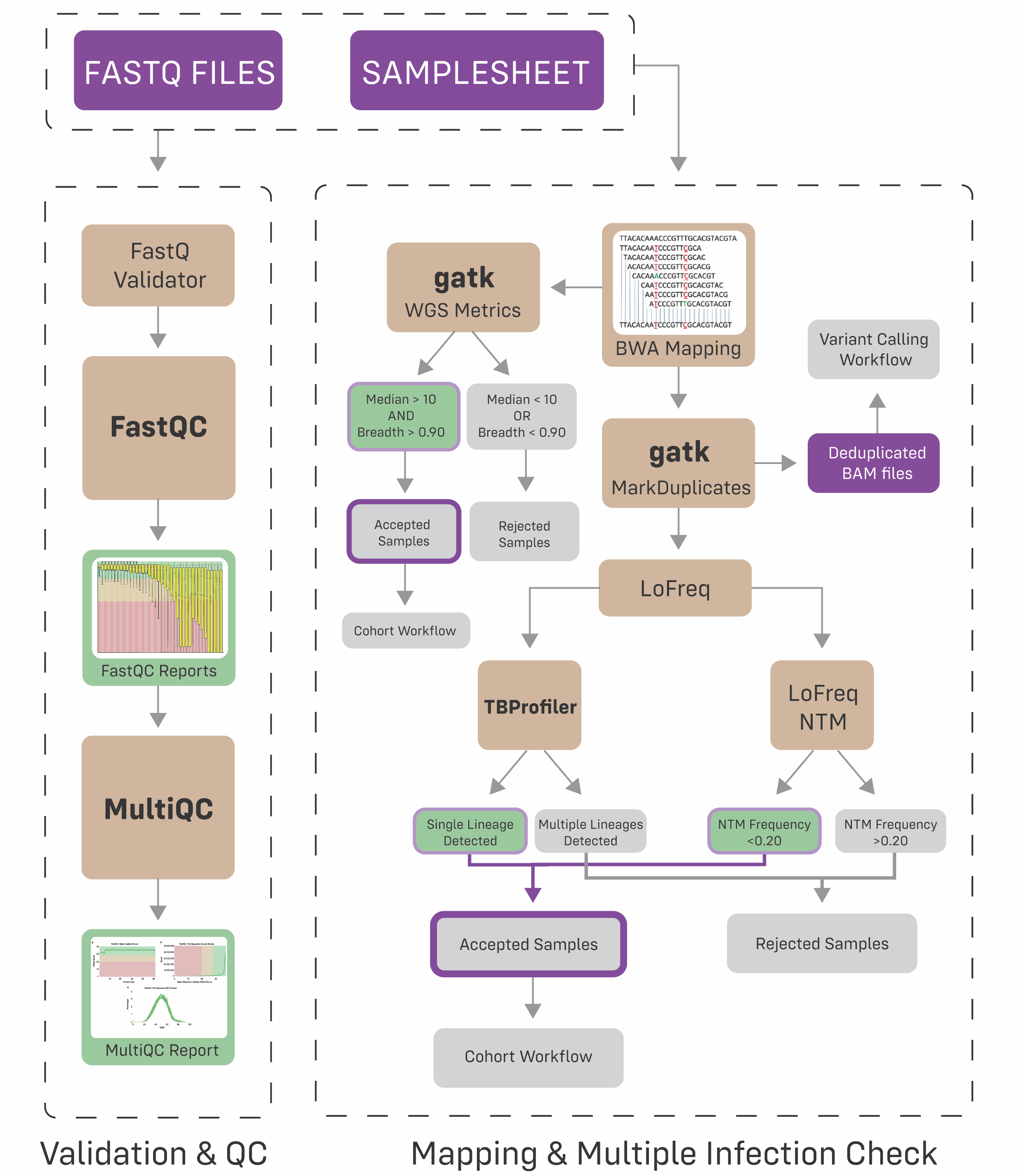


**Figure J:** Per Sample Variant Calling Workflow


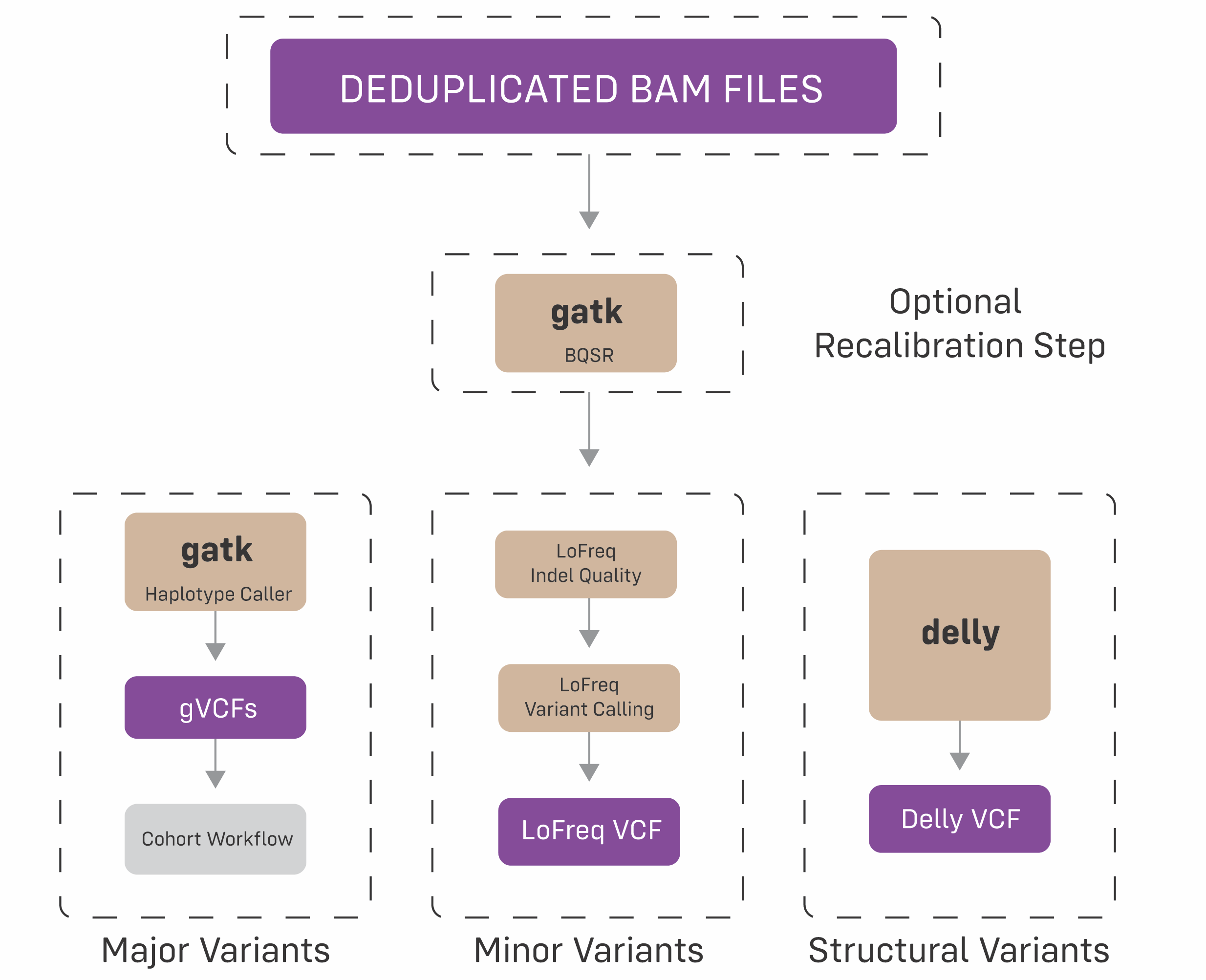


**Figure K:** Cohort Workflow


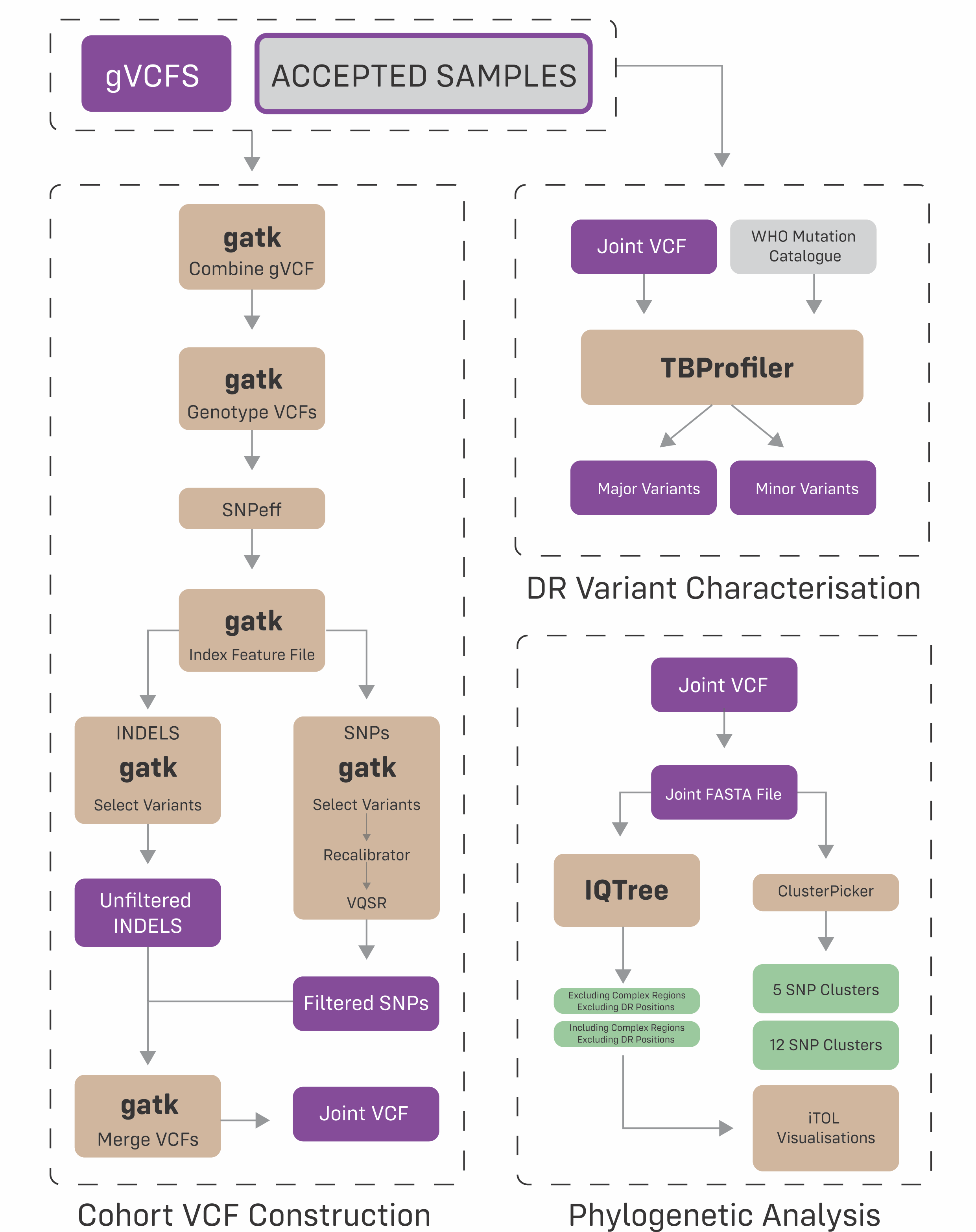

Supplement: S1 Text — (DOCX) [file pcbi.1011648.s001.docx]
